# Early life adversity predicts brain-gut alterations associated with increased stress and mood

**DOI:** 10.1016/j.ynstr.2021.100348

**Published:** 2021-05-25

**Authors:** Elena J.L. Coley, Emeran A. Mayer, Vadim Osadchiy, Zixi Chen, Vishvak Subramanyam, Yurui Zhang, Elaine Y. Hsiao, Kan Gao, Ravi Bhatt, Tien Dong, Priten Vora, Bruce Naliboff, Jonathan P. Jacobs, Arpana Gupta

**Affiliations:** a G. Oppenheimer Center for Neurobiology of Stress and Resilience, University of California, Los Angeles, USA; b David Geffen School of Medicine, University of California, Los Angeles, USA; c Department of Urology, David Geffen School of Medicine, University of California, Los Angeles, Los Angeles, CA, USA; d Vatche and Tamar Manoukian Division of Digestive Diseases, Los Angeles, CA, USA; e UCLA Microbiome Center, Los Angeles, CA, USA; f Division of Gastroenterology, Hepatology and Parenteral Nutrition, VA Greater Los Angeles Healthcare System, Los Angeles, CA, USA; g Department of Integrative Biology and Physiology, University of California, Los Angeles, Los Angeles, CA, USA; h Imaging Genetics Center, Institute for Neuroimaging and Informatics, University of Southern California, Los Angeles, CA, USA; i Laboratory of Gastrointestinal Microbiology, Nanjing Agricultural University, PR China

**Keywords:** Early life adversity, Brain-gut microbiome, Brain functional connectivity, Glutamatergic excitotoxicity, Perceived stress, Anxiety/depression, ELA, early life adversity, CNS, central nervous system, PLS-DA, partial least squares discriminant analysis, RMS, root mean squared

## Abstract

Alterations in the brain-gut system have been implicated in various disease states, but little is known about how early-life adversity (ELA) impacts development and adult health as mediated by brain-gut interactions. We hypothesize that ELA disrupts components of the brain-gut system, thereby increasing susceptibility to disordered mood. In a sample of 128 healthy adult participants, a history of ELA and current stress, depression, and anxiety were assessed using validated questionnaires. Fecal metabolites were measured using liquid chromatography tandem mass spectrometry-based untargeted metabolomic profiling. Functional brain connectivity was evaluated by magnetic resonance imaging. Sparse partial least squares-discriminant analysis, controlling for sex, body mass index, age, and diet was used to predict brain-gut alterations as a function of ELA. ELA was correlated with four gut-regulated metabolites within the glutamate pathway (5-oxoproline, malate, urate, and glutamate gamma methyl ester) and alterations in functional brain connectivity within primarily sensorimotor, salience, and central executive networks. Integrated analyses revealed significant associations between these metabolites, functional brain connectivity, and scores for perceived stress, anxiety, and depression. This study reveals a novel association between a history of ELA, alterations in the brain-gut axis, and increased vulnerability to negative mood and stress. Results from the study raise the hypothesis that select gut-regulated metabolites may contribute to the adverse effects of critical period stress on neural development via pathways related to glutamatergic excitotoxicity and oxidative stress.

## Highlights

• Early life adversity was linked to alterations in the brain-gut-microbiome system.
• Early life adversity was associated with metabolites from the glutamate pathway.
• Early life adversity was associated with functional brain connectivity alterations.
• Integrated analyses revealed increased vulnerability to negative mood and stress.
• Mechanisms involved suggest glutamatergic excitotoxicity and oxidative stress.

## 1 Introduction

Early-life adversity (ELA) is a known disruptor capable of inducing a range of developmental changes (Tomalski and Johnson, 2010), and is associated with increased vulnerability to a variety of health conditions and psychiatric disorders later in life (Shonkoff et al., 2012). Systemic changes in response to stress during critical periods include dysregulation of peripheral gene expression (Romens et al., 2015), immune function (Carpenter et al., 2010), and hormone levels (Joung et al., 2014), in addition to perturbations of the microbiome (Foster et al., 2017), all of which may contribute to and result from direct changes in the developing central nervous system (CNS). The involvement of the gut microbiome and its interactions with the brain during this early programming period remain incompletely understood. We have previously proposed that this may occur in a *bidirectional* manner: while the brain may influence alterations of gut physiology and microbiome composition and function, the resulting altered functional output from the gut microbiome may result in neuroplastic changes in the brain (Mayer, 2011; Martin et al., 2018; Osadchiy et al., 2019a). A history of ELA has been reported in conditions ranging from obesity (Osadchiy et al., 2019b) to irritable bowel syndrome (Bradford et al., 2012; Labus et al., 2017) and inflammation (Levine et al., 2015; Gupta et al., 2017a), but few studies to date have used a systems approach to investigate perturbations in the gut metabolome and the brain in humans exposed to ELA.

A primary pathway by which ELA can influence life-long trajectories is by shaping brain development (Tomalski and Johnson, 2010). Previous studies have shown that a history of ELA is associated with alterations mainly in regions of the emotion regulation and salience networks, which in turn can influence epigenetic processes related to myelination and neurogenesis (Teicher et al., 2016; Gupta et al., 2017b). These neural changes have also been implicated in hyperarousal and difficulties with emotion regulation, and later development of negative mood states (Etkin et al., 2006; Cisler et al., 2013; Stein et al., 2007). In particular, prefrontal cortex and hippocampal volumes were persistently reduced in adolescents adopted from international orphanages (Hodel et al., 2015), and female adolescents with a history of childhood maltreatment displayed altered organization of cortical networks, which mediated psychiatric outcomes (Miskovic et al., 2010). Rodent research has shown similar findings with increased resolution: for instance, maternal separation was associated with accelerated innervation of basolateral amygdala axons into the prefrontal cortex, with females specifically demonstrating reduced functional connectivity between these regions across maturation, and increased anxiety-like behavior (Honeycutt et al., 2020).

In addition to neural development, the gut is also sensitive to ELA (Dong and Gupta, 2019). A number of early developmental factors have been implicated in gut microbiome development, especially factors relating to maternal stress, diet, and disease (Zijlmans et al., 2015), mode of delivery (Yassour et al., 2016; Backhed et al., 2015), early nutrition/breast-feeding (Backhed et al., 2015; Bergstrom et al., 2014), and exposure to antibiotics (Yassour et al., 2016). In a youth cohort, early life adversity was not only associated with gastrointestinal symptoms and later anxiety, but also correlated with microbial diversity, and taxonomic abundances predicted prefrontal cortex activity (Callaghan et al., 2020). In a sample of pregnant women, early adversity correlated positively with *Prevotella*, and cortisol correlated positively with *Rikenellaceae* and *Dialister*, and negatively with *Bacteroides*, suggesting an interaction between early adversity, current stress, and gut microbiota (Hantsoo et al., 2019). Additionally, gut signaling to the brain can be mediated by metabolites produced directly by gut microbes or indirectly from host cells responding to microbial cues (Wall et al., 2014). For example, transplantation of microbiota from depressed patients into germ-free mice promoted anxiety- and depression-related behaviors compared to germ-free mice transplanted with a non-depressed microbiota; these interactions were mediated by selective modulation of both microbial and host genes involved in carbohydrate and amino acid metabolism (Zheng et al., 2016). Additionally, microbiome-derived short-chain fatty acids ameliorate stress-induced cortisol release in humans when delivered directly to the colon (Dalile et al., 2020) and ameliorate early chronic stress in rodents when delivered orally (Van de Wouw et al., 2018), further underscoring potential relationships between the brain-gut-microbiome axis and stress (Lyte et al., 2011).

While findings from animal models support a role for the gut microbiome in mediating adverse effects of ELA on neurodevelopment (De Palma et al., 2015; Moussaoui et al., 2017), comprehensive investigation of these interactions in humans is lacking. Despite the well-known limitations of cross-sectional and retrospective data, herein we test the primary hypothesis that ELA-related alterations in gut microbial metabolites are associated with alterations in brain connectivity, disordered mood, and increased vulnerability to stress in adulthood.

## 2 Materials and methods

### 2.1 Participants

The study was comprised of 128 right-handed healthy participants (43 males and 85 females), with the absence of significant medical or psychiatric conditions, as assessed by a physical exam, detailed medical history, and a clinical assessment using the modified Mini-International Neuropsychiatric Interview Plus 5.0 (MINI) (Sheehan et al., 1998). Participants were excluded for the following: pregnant or lactating, substance use, abdominal surgery, tobacco dependence (half a pack or more daily), extreme strenuous exercise (>8h of continuous exercise per week), current or past psychiatric illness, and major medical or neurological conditions. Participants taking medications that interfere with the CNS or using analgesic drugs regularly (e.g. full dose antidepressants including SSRIs, NSRIs, sedatives or anxiolytics, and opioids) were excluded. Participants were also excluded for use of antibiotics or probiotic supplements in the past 3 months. Since female sex hormones such as estrogen are known to effect brain structure and function, we requested females to stop taking hormonal contraceptives for the duration of the study. In addition, we assessed only females who were premenopausal (i.e., women under than or equal to 45 years who reported regular menses for at least 1 year) and were scanned during the follicular phase of the menstrual cycle (i.e., defined as 4–12 days after the first day of the last menstrual period), as assessed by self-report.

All procedures complied with and were approved by the Institutional Review Board (16–000187, 15–001591) at the University of California, Los Angeles's Office of Protection for Research Subjects. All participants provided written informed consent.

### 2.2 Questionnaires

ELA was measured using the Early Traumatic Inventory-Self Report (ETI-SR) (Bremner et al., 2005), a 27-item questionnaire. This questionnaire assesses the histories of childhood traumatic and adverse life events that occurred before the age of 18 years old and covers four domains: general trauma (11 items), physical punishment (5 items), emotional abuse (5 items), and sexual abuse (6 items). General traumatic events comprise a range of stressful and traumatic events that can be mostly secondary to chance events. Sample items on this scale include death of a parent, discordant relationships or divorce between parents, or death or sickness of a sibling or friend. Physical abuse involves physical contact, constraint, or confinement, with intent to hurt or injure. Sample items on the physical abuse subscale include being spanked by hand or being hit by objects. Emotional abuse is verbal communication with the intention of humiliating or degrading the victim. Sample items on the ETI-SR emotion subscale include the following, “Often put down or ridiculed,” or “Often told that one is no good.” Sexual abuse is unwanted sexual contact performed solely for the gratification of the perpetrator or for the purposes of dominating or degrading the victim. Sample items on the sexual abuse scale include being forced to pose for suggestive photographs, to perform sexual acts for money, or coercive anal sexual acts against one's will. The ETI-SR instrument was chosen due to its psychometric properties, ease of administration, time efficiency, and ability to measure ELAs in multiple domains (Bremner et al., 2007). For subsequent analyses, participants were split into two groups: “High ETI” (ETI-SR total > 4) and “Low ETI” (ETI-SR total ≤ 4). This cut off ETI score was selected based on the median score of this sample versus the mean ETI score in past papers because of the presence of extreme ETI scores in the data. While some studies have reported a higher mean ETI, our cut-off falls in line with previously reported healthy patient samples tested using the short-form version of the tool (mean = 3.5, sd = 3.3^39^; mean = 2.68, sd = 2.55^40^).

Additional questionnaires included the Perceived Stress Scale (PSS) and the Hospital Anxiety and Depression Scale (HADS). The PSS is a 10-item scale used to measure stressful demands in a given situation, indicating that demands exceed ability to cope (Cohen et al., 1983). The questions are based on subjects reporting the frequency of their feelings within the past month to each question, which are scored on a scale of 0 (never) to 4 (very often). The HADS is a 14-item scale used to measure symptoms of anxiety and depression (Zigmond and Snaith, 1983). The questions are scored on a scale of 0–3, corresponding to how much the individual identifies with the question for the past week.

Diet was assessed through a self-reported UCLA Diet Checklist, is a questionnaire developed by our institution, intended to represent the diet that best reflects what patients consume on a regular basis. The specific diets incorporated into this checklist include the following options: i) Standard American (characterized by high consumption of processed, frozen, and packaged foods, pasta and breads, and red meat; vegetables and fruits are not consumed in large quantities), ii) Modified American (high consumption of whole grains including some processed, frozen, and packaged foods; red meat is consumed in limited quantities; vegetables and fruit are consumed in moderate to large quantities), iii) Mediterranean (high consumption of fruits, vegetables, beans, nuts, and seeds; olive oil is the key monounsaturated fat source; dairy products, fish, and poultry are consumed in low to moderate amounts and little red meat is eaten), and iv) all other diets that do not fit into the above categories. If they marked “other” they were asked to describe the components of their individual diet with regards to consumption of meat, dairy, eggs, fruits, vegetables, and grains. If a participant selected “other”, their comments regarding intake of food components were individually reviewed, as was that participant's previous 24-h food intake. Our institution's Diet Checklist was then internally validated against the standardized DHQ-III. For data analysis we had 3 diet categories: We combined standard American and modified American diet as one category. Mediterranean, vegan, vegetarian, and gluten-free were combined into a single category, and all other diets were combined as “other.” For the analyses, the three categories (America, Mediterranean/Plant based, Other) were used.

### 2.3 Gut microbiome

#### 2.3.1 Collection and storage

These have been previously described in great detail in recently published papers (Dong et al., 2020a, [Bibr bib28][Bibr bib27][Bibr bib28]; Osadchiy et al., 2020). Participants were given “at home collection kits” which consisted of a standard laboratory supplies such as collection hat over the commode and a urine cup to pack the fresh stool. The participants were given specific instructions regarding time of stool collection (e.g., time of day and within 2–3 days before the MRI scan). In addition, 2–3 consecutive diet diaries were collected from the time of enrollment to the time of the MRI scan and stool collection (1 weekday and 1 weekend). Participants were asked to collect the stool before the first meal of the day. If participants were on antidiarrheal or laxatives, they were asked to refrain from use for 2–3 days before the sample collection. Participants were asked to store their fresh stool immediately in the freezer immediately upon collection and to bring in the stool to the laboratory on the day of the MRI (note day and time of stool collection and storage). Any deviation from the stool sample collection or storage were documented in order to account for in the analyses. Fecal samples were stored at −80 °C, then ground into a coarse powder by mortar and pestle under liquid nitrogen and aliquoted for DNA extraction and metabolomic profiling.

#### 2.3.2 Fecal microbial profiling

DNA extraction with bead beating was performed using the QIAGEN Powersoil DNA Isolation Kit (MO BIO Laboratories, Carlsbad, CA) with bead beating following the manufacturers protocol. The V4 hypervariable region of the 16S rRNA gene was then amplified using the 515F and 806R primers to generate a sequencing library according to a published protocol (Tong et al., 2014). The PCR products were purified with a commercial kit. The library underwent 2 × 250 sequencing on an Illumina HiSeq 2500 to a mean depth of 250,000 merged sequences per sample. QIIME 2 2021.2.0 was used to perform quality filtering, merge paired end reads, and cluster sequences into 97% operational taxonomic units (OTUs) (Caporaso et al., 2010). OTUs were classified taxonomically using the SILVA (128 release) database at the level of domain, phylum, family, genus, and species, depending on the depth of reliable classifier assignments.

Microbial alpha diversity was assessed on datasets rarefied to equal sequencing depth (34,222) using the Chao1 index of richness and the Shannon index of evenness. Microbial composition was compared across samples by Microbial composition was compared across samples by using the DEICODE plugin in QIIME 2 which employs a robust Aitchison distance metric. This beta diversity metric accounts for the sparse compositional nature of the microbiome, which has a demonstratively higher discriminatory power compared to other distance metrics such as UniFrac and Bray-Curtis (Anderson, 2001). These differences were visualized with principal coordinates analysis. The significance of differences in microbial composition between individuals with high or low ETI scores, adjusting for age, BMI, diet, and sex was assessed using PERMANOVA with 100,000 permutations (Anderson, 2001). Differential abundance of microbial genera was determined using multivariate negative binomial mixed models implemented in DESeq2 that included age, BMI, diet, and sex as covariates (Love et al., 2014). P-values were adjusted for multiple hypothesis testing to generate q-values, with a significance threshold of q < 0.05.

#### 2.3.3 Fecal metabolomic profiling

Fecal aliquots were shipped to Metabolon, Inc., and run as a single batch on their global HD4 metabolomics platform (Evans et al., 2009). This involved running methanol extracted samples through ultrahigh performance liquid chromatography-tandem mass spectroscopy under four separate chromatography and electrospray ionization conditions to separate compounds with a wide range of chemical properties. Compounds were identified by comparison of spectral features to Metabolon's proprietary library that includes MS/MS spectral data on more than 3300 purified standards. Study specific technical replicates generated by pooling aliquots of all samples were used to measure total process variability (median relative standard deviation 13%). Results were provided as scaled, imputed abundances of 872 known compounds.

Missing values of raw data were filled up using median values, and ineffective peaks were removed through the interquartile range denoising method. In addition, the internal standard normalization method was employed in the data analysis. The dataset for the multiple classification analysis was compiled from the metabolite profiling results and a 3D matrix involving metabolite numbers, sample names, and normalized peak intensities were fed into the MetaboAnlyst web software 3.0 (http://www.metaboanalyst.ca).

### 2.4 Magnetic resonance imaging

Whole brain structural and functional (resting state) data was acquired using a 3.0T Siemens Prisma MRI scanner (Siemens, Erlangen, Germany). Detailed information on the standardized acquisition protocols, quality control measures, and image preprocessing are provided in previously published studies (Gupta et al., 2017b; Dong et al., 2020b; Osadchiy et al., 2020).

#### 2.4.1 Structural MRI acquisition

High resolution T1-weighted images were acquired: echo time/repetition time (TE/TR) = 3.26 ms/2200 ms, field of view = 220 × 220 mm slice thickness = 1 mm, 176 slices, 256 × 256 voxel matrices, and voxel size = 0.86 × 0.86 × 1 mm.

#### 2.4.2 Functional MRI acquisition

Resting-state scans were acquired with eyes closed and an echo planar sequence with the following parameters: TE/TR = 28 ms/2000 ms, flip angle = 77°, scan duration = 8m6s–10m6s, FOV = 220 mm, slices = 40 and slice thickness = 4.0 mm, and slices were obtained with whole-brain coverage.

#### 2.4.3 Preprocessing of MRI images

Preprocessing and quality control of functional images was done using SPM-12 software (Welcome Department of Cognitive Neurology, London, UK). The first two volumes were discarded to allow for stabilization of the magnetic field. Slice timing correction was performed first, followed by rigid six-degree motion-correction for the six realignment parameters. The motion correction parameters in each degree were examined for excessive motion. If any motion was detected above 2 mm translation or 2° rotation, the scan, along with the paired structural scan was discarded. In order to robustly take account the effects of motion, root mean squared realignment estimates were calculated as robust measures of motion using publicly available MATLAB code. Any subjects with a greater RMS value than 0.25 was not included in the analysis. The resting state images were then co-registered to their respective anatomical T1 images. Each T1 image was then segmented and normalized to a smoothed template brain in Montreal Neurological Institute (Melander et al., 1999) template space. Each subject's T1 normalization parameters were then applied to that subject's resting state image, resulting in an MNI space normalized resting state image. The resulting images were smoothed with 5 mm (Romens et al., 2015) Gaussian kernel. For each subject, a sample of the volumes was inspected for any artifacts and anomalies. Levels of signal dropout were also visually inspected for excessive dropout in a priori regions of interest.

#### 2.4.4 Structural image parcellation

T1-image segmentation and cortical and subcortical regional parcellation were conducted using Schaefer 400 atlas (Schaefer et al., 2018), Harvard-Oxford subcortical atlas (Destrieux et al., 2010), and the Ascending Arousal Network atlas (Edlow et al., 2012). This parcellation results in the labeling of 430 regions, 400 cortical structures, 14 bilateral subcortical structures, bilateral cerebellum, and 14 brainstem nuclei.

#### 2.4.5 Functional brain connectivity matrix construction

To summarize, all pre-processed, normalized images were entered into the CONN-fMRI functional connectivity toolbox version 17 in MATLAB. All images were first corrected for noise using the automatic component-based noise correction (aCompCor) method to remove physiological noise without regressing out the global signal. Confounds for the six motion parameters along with their first-order temporal derivatives, along with confounds emerging from white matter and cerebral spinal fluid, and first-order temporal derivatives of motion, and root mean squared values of the detrended realignment estimates were removed using regression. Although the influence of head motion cannot be completely removed, CompCor has been shown to be particularly effective for dealing with residual motion relative to other methods. The images were then band-pass filtered between 0.008 and 0.009 Hz to minimize the effects of low frequency drift and high frequency noise after CompCor regression. Connectivity matrices for each subject, consisting of all the parcellated regions were then computed. This represents the association between two average temporal BOLD time series across all the voxels in each region. The final outputs for each subject consisted of a connectivity matrix between the 430 parcellated regions and was indexed by Fisher transformed Z correlation coefficients between each region of interest.

### 2.5 Statistical analysis

#### 2.5.1 Sparse partial least squares — discriminate analysis

A partial least squares-discriminant analysis (PLS-DA) was conducted in R (Boston, MA) to explore the group difference between high vs. low ETI groups by incorporating known classifications for the metabolites. Similarly, a sparse PLS-DA for whole brain resting state connectivity was run to understand the classification in brain signatures related to high vs. low ETI. In order to prevent overfitting of the model, we ran permutation tests. The metabolites with values of the first component of variable importance projection (VIP) greater than 1.0 were assessed, indicating the estimate of the importance of each metabolite used in the model. The brain connectivity regions/brain signatures from the two components of the weighted design matric and contributing to the discrimination between the two groups were summarized using the top variable loadings on the individual dimensions/components and VIP coefficients. T-tests using contrasts in a general linear model controlling for age, BMI, diet (3 categories), and sex (male, female) were conducted. *P-*values were adjusted for with the Benjamini-Hochberg false discovery rate (FDR) procedure and significant *q*-values, were reported (Benjamini and Hochberg, 1995). The metabolites with VIPs >1.0 and *q* < 0.05 were selected as significantly different between the two groups. The fold change was also calculated to investigate the difference by comparing the mean value of the peak area obtained between the two groups.

#### 2.5.2 Network analysis

Network analysis was performed to integrate information from three data sets:

1) stool-derived metabolites 2) clinical data (ETI, PSS, HADS Anxiety, HADS Depression) and 3) functional connectivity brain data. The interaction between the phenome (clinical measures), microbiome (stool-derived metabolites) and connectome (brain connectivity) was determined by computing Spearman correlations between different data types in R v. 3.6.2, controlling for age, BMI, diet, and sex. These correlations were run separately for 1. All participants 2. the low ETI group and for 3. the high ETI group. Circos images were created to visualize and construct brain, symptom, and gut-derived metabolite interaction networks thresholded at FDR corrected *q* < 0.05. We present the networks by placing nodes of the same type of data together and displaying connecting edges representing correlations. A red edge indicates a significant correlation in the high ETI group. A green edge indicates a significant correlation in the low ETI group. A grey edge represents a significant correlation for all the participants as a function of increasing ETI scores.

## 3 Results

### 3.1 Subject demographics and clinical variables

Individuals in the low ETI group had a mean score of 1.2, while those in the high ETI group had a mean score of 8.6. Those with a history of high ELA exposure as indexed by the ETI scale had significantly higher BMI (p < 0.001) and anxiety (p = 0.032) levels (Table 1). Although the high ELA group was older (p = 0.244), and reported higher levels of depression symptoms (p = 0.284), and perceived stress (p = 0.069), these differences were not significant. To account for the significant difference in BMI between low and high ETI groups, we controlled for it in subsequent multivariate analyses. Diet did not differ by ETI group (American: High ETI = 15, Low ETI = 19, Mediterranean/Plant Based: High ETI = 25, Low ETI = 39, Other: High ETI = 12, Low ETI = 18.

**Table 1:** Clinical characteristics associated with early life adversity.

Psychological Measures	Low ETI		N	High ETI		N	t-value	p-value
	Mean	SD		Mean	SD			
**Sex**	48 Female	28 Male	76	37 Female	15 Male	52	–	–
**Age**	27.28	7.55	76	28.81	7.051737	52	−1.17	0.2441
**ETI**	1.20	1.36	76	8.60^a^	3.40	52	16.27	**0.0001**
**BMI (kg/m**^**2**^**)**	27.42	5.16	76	30.76	5.30	52	−3.53	**0.0006**
**PSS Score**	11.18	6.39	76	13.37	6.77	52	−1.84	0.0690
**HADS Anxiety**	3.97	3.33	76	5.33	3.55	52	−2.17	**0.0322**
**HADS Depression**	1.82	2.00	76	2.29	2.69	52	−1.08	0.2845

N = 128 total, Low ETI group N = 76, High ETI group N = 52.

Means and standard deviations are reported for normally distributed data. ETI threshold = 4 (Low ETI: ETI-SR total ≤ 4, High ETI: ETI-SR total > 4). ETI: early traumatic inventory; BMI: body mass index, PSS: Perceived Stress Scale, HADS: Hospital Anxiety and Depression Scale.

p-significant <0.05.

a Previous reports for mean ETI in healthy adults are 7.5 (sd 5.4)^41^.

### 3.2 Early life adversity and gut microbiome composition

There were no significant relationships between a history of ELA exposure and microbial alpha diversity, the variation of microbes within a sample, (Chao1, p = 0.345; Shannon, p = 0.465) (Supplementary Fig. 1A), microbial beta diversity, the variation of microbial communities between samples, (measured by Aitchison-based PCoA; permanova p = 0.421) (Supplementary Fig. 1B) or relative taxonomic abundance, at either the phylum or genus levels (Supplementary Fig. 1C).

### 3.3 Early life adversity associates with adult gut metabolites

**Fig. 1:**
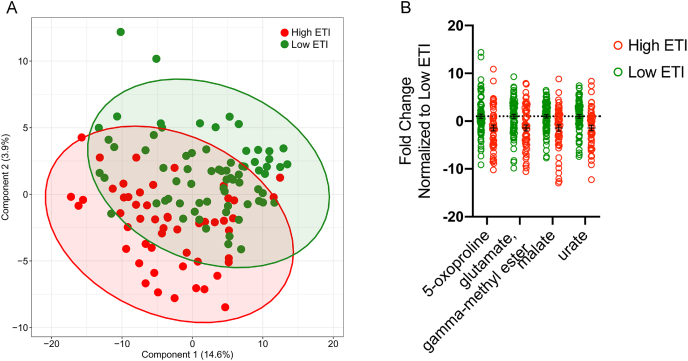
**Early Life Adversity Differentiates Fecal Metabolite Composition**. N = 128 total, Low ETI group N = 76, High ETI group N = 52. A: Gut metabolites cluster by PLS-DA. B: Fold change of significant metabolites after FDR correction, q < 0.05. Errors bars represent mean ± SEM.

The PLS-DA of the gut metabolites showed a defined clustering, based on low or high ETI exposure (Table 2; Fig. 1A). Out of 557 gut metabolites screened, 207 loaded on component 1 > 1.0, and were classified as “VIP” metabolites. Of this narrowed-down list of 207, 33 metabolites showed a significant relationship to ETI exposure (p < 0.05), belonging to amino acid, carbohydrate, cofactors and vitamins, energy, lipid, nucleotide, and xenobiotics super pathways (Table 2). After correcting for multiple comparisons, four metabolites remained significantly correlated with ETI exposure: glutamate, gamma-methyl ester (p = 0.022, q = 0.044), in the glutamate metabolism sub-pathway; 5-oxoproline (p = 0.020, q = 0.044), in the glutathione metabolism sub-pathway; malate (p = 0.003, q = 0.018), in the tricarboxylic acid (TCA) cycle sub-pathway; and urate (p = 0.002, q = 0.036), in the purine metabolism sub-pathway. Of these four significant metabolites, all were reduced by approximately two-fold in individuals with high ETI exposure, compared to those with low ETI exposure (Fig. 1B).

**Table 2:** Gut metabolites associated with early life adversity.

VIP metabolites	Super Pathway	Sub Pathway	beta	se	t	p-value	q-value
glutamate, gamma-methyl ester	Amino Acid	Glutamate Metabolism	−0.407	0.175	−2.322	**0.022**	**0.044**
5-oxoproline	Amino Acid	Glutathione Metabolism	−0.425	0.180	−2.356	**0.020**	**0.044**
formiminoglutamate	Amino Acid	Histidine Metabolism	−0.424	0.184	−2.308	**0.023**	0.219
N6-formyllysine	Amino Acid	Lysine Metabolism	0.423	0.188	2.254	**0.026**	0.219
N,N,N-trimethyl-5-aminovalerate	Amino Acid	Lysine Metabolism	−0.462	0.185	−2.501	**0.014**	0.219
N,N-dimethyl-5-aminovalerate	Amino Acid	Lysine Metabolism	−0.519	0.184	−2.822	**0.006**	0.219
N6,N6-dimethyllysine	Amino Acid	Lysine Metabolism	0.413	0.189	2.190	**0.030**	0.219
N1,N12-diacetylspermine	Amino Acid	Polyamine Metabolism	0.419	0.184	2.278	**0.024**	0.219
(N(1) + N(8))-acetylspermidine	Amino Acid	Polyamine Metabolism	0.402	0.185	2.177	**0.031**	0.219
diacetylspermidine*	Amino Acid	Polyamine Metabolism	0.468	0.186	2.513	**0.013**	0.219
lactate	Carbohydrate	Glycolysis, Gluconeogenesis, and Pyruvate Metabolism	−0.390	0.185	−2.102	**0.038**	0.401
xanthopterin	Cofactors and Vitamins	Pterin Metabolism	−0.410	0.186	−2.204	**0.029**	0.250
malate	Energy	TCA Cycle	−0.550	0.182	−3.016	**0.003**	**0.018**
tricarballylate	Energy	TCA Cycle	−0.378	0.188	−2.016	**0.046**	0.138
N-behenoyl-sphingadienine (d18:2/22:0)*	Lipid	Ceramides	−0.420	0.188	−2.232	**0.027**	0.282
LAHSA (18:2/OH-18:0)*	Lipid	Fatty Acid Hydroxyl Fatty Acid	−0.405	0.184	−2.198	**0.029**	0.282
dihydroorotate	Lipid	Fatty Acid Metabolism(Acyl Carnitine)	−0.485	0.187	−2.598	**0.011**	0.234
azelate (nonanedioate; C9)	Lipid	Fatty Acid, Dicarboxylate	−0.396	0.188	−2.102	**0.038**	0.282
maleate	Lipid	Fatty Acid, Dicarboxylate	−0.499	0.187	−2.671	**0.009**	0.234
mevalonate	Lipid	Mevalonate Metabolism	0.406	0.183	2.223	**0.028**	0.282
1-palmitoylglycerol (16:0)	Lipid	Monoacylglycerol	0.405	0.185	2.186	**0.031**	0.282
pregnen-diol disulfate*	Lipid	Pregnenolone Steroids	0.542	0.187	2.902	**0.004**	0.235
lithocholic acid sulfate (2)	Lipid	Secondary Bile Acid Metabolism	0.367	0.182	2.017	**0.045**	0.282
sphinganine	Lipid	Sphingolipid Synthesis	−0.384	0.188	−2.042	**0.043**	0.282
allantoin	Nucleotide	Purine Metabolism, (Hypo)Xanthine/Inosine containing	−0.423	0.185	−2.290	**0.024**	0.148
urate	Nucleotide	Purine Metabolism, (Hypo)Xanthine/Inosine containing	−0.569	0.182	−3.122	**0.002**	**0.036**
pseudouridine	Nucleotide	Pyrimidine Metabolism, Uracil containing	−0.376	0.185	−2.028	**0.045**	0.148
3-(3-hydroxyphenyl)propionate	Xenobiotics	Benzoate Metabolism	0.467	0.182	2.560	**0.012**	0.107
3-(4-hydroxyphenyl)propionate	Xenobiotics	Benzoate Metabolism	0.389	0.188	2.071	**0.040**	0.141
3,4-dihydroxybenzoate	Xenobiotics	Benzoate Metabolism	−0.395	0.179	−2.212	**0.029**	0.121
piperidine	Xenobiotics	Food Component/Plant	−0.459	0.187	−2.459	**0.015**	0.107
sitostanol	Xenobiotics	Food Component/Plant	−0.489	0.186	−2.630	**0.009**	0.107
sucralose	Xenobiotics	Food Component/Plant	−0.416	0.188	−2.218	**0.028**	0.121

N = 128 total, Low ETI group N = 76, High ETI group N = 52.

p-value significant <0.05.

Q-values derived from FDR correction, q-value significant <0.05.

### 3.4 Early life adversity associates with brain functional connectivity

**Fig. 2:**
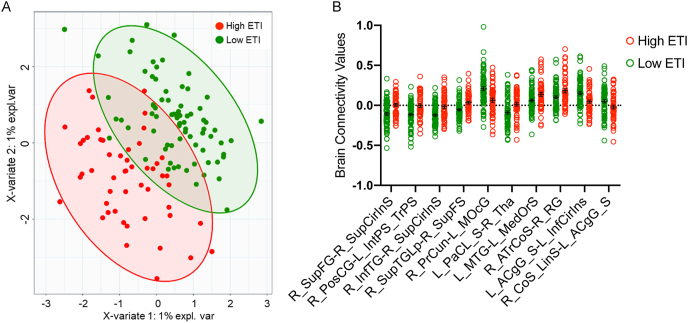
Early Life Adversity Differentiates Brain Connectivity. N = 128 total, Low ETI group N = 76, High ETI group N = 52. A: Brain connectivity clusters by SPLS-DA. B: Significant regions after FDR correction, q < 0.05. Error bars represent mean ± SEM.

**Fig. 3:**
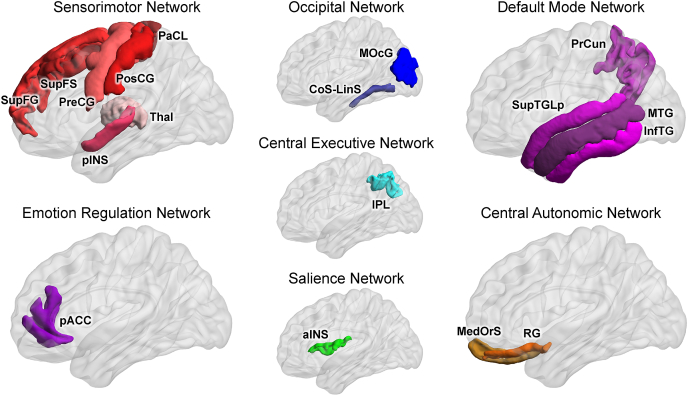
**Early Life Adversity Impacts Multiple Brain NetworksBrain Regions**: SupFG/S: superior frontal gyrus and sulcus, PreCG: precentral gyrus, PostCG: postcentral gyrus, PaCL: paracentral lobule, pINS: posterior insula; Thal: thalamus, pACC: pregenual anterior cingulate cortex, MOcG: middle occipital gyrus, CoS-LinS: medial occipito-temporal sulcus (collateral sulcus) and lingual sulcus, IPL: inferior parietal lobule, aINS: anterior insula, PrCun: precuneus, SupTGLp: lateral aspect of the superior temporal gyrus, MTG: middle temporal gyrus, InfTG: inferior temporal gyrus, MedOrS: medial orbital sulcus (olfactory sulcus), RG: gyrus rectus (straight gyrus).

A sPLS-DA of brain functional connectivity displayed significant clustering based on low or high ETI exposure (Table 3; Fig. 2A). Connectivity between eleven pairs of brain regions were significantly associated with ETI exposure (p < 0.05), and after correcting for multiple comparisons, ten pairs of regions remained significant (q < 0.05) (Table 3). All regions found to be significantly different have been summarized in Fig. 3 (represented by regions in each specific brain network).

**Table 3:** Brain connectivity associated with early life adversity.

Network A	Region A	Network B	Region B	LOADINGS Comp 1	LOADINGS Comp 2	VIP Comp 1	VIP Comp 2	t	p-value	q-value	Interpretation
Brain Signature 1											
SMN	R_SupFG (SMA)	SAL	R_SupCirInS (aINS)	−0.778		236.196	164.545	−4.271	**3.80E-05**	**2.99E-04**	high ETI ↑
SMN	R_PosCG (SI)	CEN	L_IntPS_TrPS (IPL)	−0.467		141.889	98.846	−4.149	**6.10E-05**	**2.99E-04**	high ETI ↑
DMN	R_InfTG (LTC)	SAL	R_SupCirInS (aINS)	−0.395		119.872	83.508	−4.120	**6.80E-05**	**2.99E-04**	high ETI ↑
DMN	R_SupTGLp (LTC)	SMN	R_SupFS (SMA)	−0.145		44.041	30.681	−4.023	**9.83E-05**	**2.99E-04**	high ETI ↑
DMN	R_PrCun	OCC	L_MOcG (OCC)	0.011		3.181	78.003	3.971	**1.19E-04**	**2.99E-04**	low ETI ↑
**Brain Signature 2**											
SMN	L_PaCL_S	SMN	R_Thal		−0.660		143.839	−3.353	**0.001**	**0.002**	high ETI ↑
DMN	L_MTG (LTC)	CAN	L_MedOrS (OFC)		−0.434		94.628	−2.525	**0.013**	**0.019**	high ETI ↑
DMN	R_ATrCoS (LTC)	CAN	R_RG (OFC)		−0.168		36.603	−2.674	**0.009**	**0.014**	high ETI ↑
DMN	R_PrCun	SAL	R_SupCirInS (aINS)		0.199		43.528	1.562	0.121	0.121	low ETI ↑
DMN	R_PrCun	SMN	L_PRCG (M1)		0.251		54.791	2.046	**0.043**	0.054	low ETI ↑
DMN	R_PrCun	SMN	R_PosCG (S1)		0.162		35.258	1.649	0.102	0.117	low ETI ↑
DMN	R_PrCun	SMN	L_PRCG (M1)		0.096		20.959	1.567	0.120	0.121	low ETI ↑
DMN	R_PrCun	OCC	L_MOcG (OCC)		0.358	3.181	78.003	3.971	**1.19 E-04**	**2.99 E-04**	low ETI ↑
ERN	L_ACgG_S (pACC)	SMN	L_InfCirInS (aINS)		0.277		60.415	3.583	**4.84 E-04**	**1.04E-04**	low ETI ↑
OCC	R_CoS_LinS (OCC)	ERN	L_ACgG_S (pACC)		0.062		13.483	2.329	**0.021**	**0.029**	low ETI ↑

N = 128 total, Low ETI group N = 76, High ETI group N = 52.

p-value significant <0.05.

Q-values derived from FDR correction, q-value significant <0.05.

Comp: Components; VIP: variable importance projection.

Networks.

SMN: sensorimotor, DMN: default mode, SAL: salience, CEN: central executive, CAN: central autonomic, ERN: emotion regulation, OCC: occipital.

Brain Regions.

SupFG: superior frontal gyrus, SMA: supplementary motor area, SupCirInS: superior segment of the circular sulcus of the insula, aINS: anterior insula, PosCG: postcentral gyrus, S1: primary somatosensory cortex, IntPS_TrPS: intraparietal sulcus (interparietal sulcus) and transverse parietal sulci, IPL: inferior parietal lobule, InfTG: inferior temporal gyrus, LTC: lateral temporal cortex, SupTGLp: lateral aspect of the superior temporal gyrus, SupFS: superior frontal sulcus, MOcG: middle occipital gyrus, OCC: occipital lobe, PaCL/S: paracentral lobule and sulcus, Thal: thalamus, MTG: middle temporal gyrus, MedOrS: medial orbital sulcus (temporal sulcus), OFC: orbitofrontal cortex, ATrCoS: anterior transverse collateral sulcus, RG: straight gyrus (gyrus rectus), PrCun: precuneus, PRCG: precentral gyrus, ACgG_S: anterior part of the cingulate gyrus and sulcus, pACC: pregenual anterior cingulate cortex, InfCirInS: inferior segment of the circular sulcus of the insula, CoS_LinS: medial occipito-temporal sulcus (collateral sulcus) and lingual sulcus.

High ETI-related connectivity was observed between salience, sensorimotor, central executive, default mode and central autonomic networks. Specific positive relationships included salience (superior segment of the circular sulcus of the insula) with both sensorimotor (superior frontal gyrus (q < 0.001)) and default mode (inferior temporal gyrus (q < 0.001)); sensorimotor (post-central gyrus) with central executive (intraparietal sulcus, interparietal sulcus, and transverse parietal sulci (q < 0.001)); default mode (lateral aspect of the superior temporal gyrus) with sensorimotor (superior frontal sulcus (q < 0.001)); sensorimotor (paracentral lobule and sulcus) with sensorimotor (thalamus (q = 0.002)); default mode (middle temporal gyrus and anterior transverse collateral sulcus) with central autonomic (medial orbital sulcus (q = 0.019) and straight gyrus (gyrus rectus) (q = 0.014), respectively) (Fig. 2B; Fig. 3; Table 3).

Low ETI-related connectivity was observed between occipital, default mode, and emotion regulation networks, including: default mode (precuneus) with occipital (middle occipital gyrus (q < 0.001)); emotion regulation (anterior part of the cingulate gyrus and sulcus) with both sensorimotor (inferior segment of the circular sulcus of the insula (q < 0.001)) and occipital (medial occipito-temporal sulcus (collateral sulcus) (q = 0.029)). (Fig. 2B; Fig. 3; Table 3). Additionally, low ETI exposure correlated with increased connectivity approaching significance between default mode (precuneus) and sensorimotor (precentral gyrus (q = 0.054)).

### 3.5 Early life adversity correlates with alterations in the brain-gut-microbiome system and current mood symptoms

**Fig. 4:**
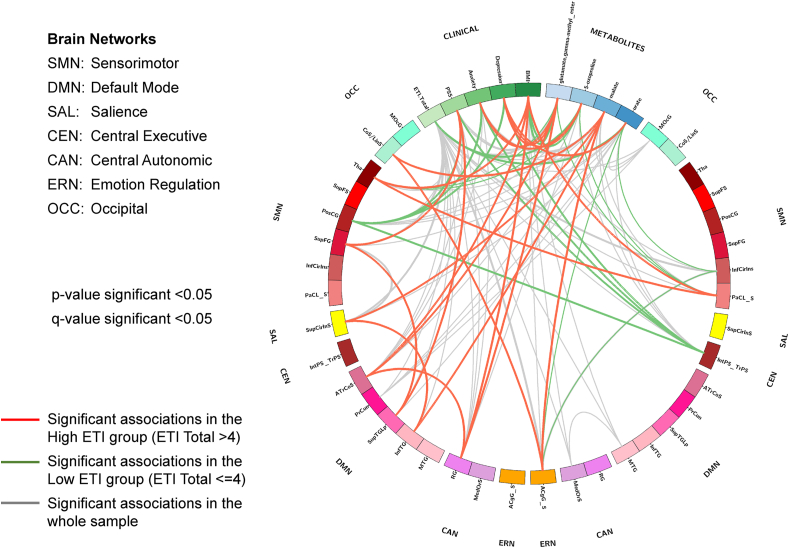
Early Life Adversity Interacts with Clinical Variables, Gut Metabolites and Brain Connectivity N = 128 total, Low ETI group N = 76, High ETI group N = 52. p-value significant <0.05. Q-values derived from FDR correction, q-value significant <0.05. Red Line: Significant associations in the High ETI group (ETI Total >4). Green Line: Significant associations in the High ETI group (ETI Total<=4). Grey Line: Significant associations in the whole sample. **Networks**: SMN: sensorimotor, DMN: default mode, SAL: salience, CEN: central executive, CAN: central autonomic, ERN: emotion regulation, OCC: occipital. Brain Regions: SupFG: superior frontal gyrus, SupCirInS: superior segment of the circular sulcus of the insula, PosCG: postcentral gyrus, IntPS_TrPS: intraparietal sulcus (interparietal sulcus) and transverse parietal sulci, InfTG: inferior temporal gyrus, SupTGLp: lateral aspect of the superior temporal gyrus, SupFS: superior frontal sulcus, PaCL/S: paracentral lobule and sulcus, Thal: thalamus, ATrCoS: anterior transverse collateral sulcus, RG: straight gyrus (gyrus rectus), ACgG_S: anterior part of the cingulate gyrus and sulcus, InfCirInS: inferior segment of the circular sulcus of the insula; CoS_LinS: medial occipito-temporal sulcus (collateral sulcus) and lingual sulcus. Clinical Variables: ETI: early traumatic inventory; BMI: body mass index, PSS: Perceived Stress Scale, HADS: Hospital Anxiety and Depression Scale. (For interpretation of the references to colour in this figure legend, the reader is referred to the Web version of this article.)

Significant relationships were identified between the significant pairs of connected brain regions (Section 4.3), four metabolites (glutamate gamma-methyl ester, 5-oxoproline, malate, and urate; Section 4.2), and four clinical variables (ETI score, PSS score, HADS anxiety, and HADS depression; Section 4.1) (Table 4; Fig. 4). Significant associations between the variables and by ETI exposure are depicted by group (high ETI and low ETI) and after correcting for multiple comparisons (q < 0.05).

**Table 4:** Early life adversity interacts with clinical variables, gut metabolites and brain connectivity.

HIGH ETI (ETI-SR total > 4)					
			correlation coefficient	p-value	q-value
Brain x Metabolites					
R_InfTG - R_SupCirinS	DMN-SAL	malate	−0.292	0.003	**0.003**
R_ ATrCoS (LTC) - R_RG (OFC)	DMN-CAN	glutamate, gamma-methyl ester	−0.399	0.001	**0.003**
R_CoS_LinS - L_ACgG_S	OCC-ERN	malate	−0.279	0.001	**0.003**
**Brain x Clinical Variables**					
R_SupTGLp - R_SupFS	DMN-SMN	PSS Score	0.348	0.001	**0.002**
L_PaCL_S - R_Tha	SMN-SMN	BMI	−0.283	0.004	**0.002**
R_ATrCoS - R_RG	DMN-CAN	BMI	0.329	0.001	**0.004**
**Metabolites x Clinical Variables**					
HADS Anxiety		glutamate, gamma-methyl ester	−0.311	0.002	**0.005**
HADS Depression		glutamate, gamma-methyl ester	−0.299	0.003	**0.005**
HADS Depression		5-oxoproline	−0.322	0.020	**0.005**
HADS Anxiety		5-oxoproline	−0.289	0.003	**0.020**
HADS Depression		urate	−0.353	0.010	**0.013**

**LOW ETI (ETI-SR total<=4)**					
			**correlation coefficient**	**p-value**	**q-value**
**Brain x Metabolites**					
L_PaCL_S - R_Tha	SMN-SMN	glutamate, gamma-methyl ester	−0.228	0.004	**0.004**
L_PaCL_S - R_Tha	SMN-SMN	5-oxoproline	−0.229	0.004	**0.004**
L_ACgG_S - L_InfCirIns	ERN-SMN	urate	−0.229	0.004	**0.004**
**Brain x Clinical Variables**					
R_PosCG - L_IntPS_TrPS	SMN-CEN	PSS Score	0.254	0.002	**0.003**
R_PosCG - L_IntPS_TrPS	SMN-CEN	HADS Anxiety	0.264	0.002	**0.003**
R_PosCG - L_IntPS_TrPS	SMN- CEN	HADS Depression	0.333	0.003	**0.003**
**Metabolites x Clinical Variables**					
BMI		glutamate, gamma-methyl ester	−0.291	0.011	**0.022**
ETI Total Score		5-oxoproline	0.253	0.023	**0.023**
BMI		5-oxoproline	−0.266	0.021	**0.023**
ETI Total Score		urate	0.276	0.001	**0.004**

N = 128 total, Low ETI group N = 76, High ETI group N = 52.

p-value significant <0.05.

Q-values derived from FDR correction, q-value significant <0.05.

**Networks**.

SMN: sensorimotor, DMN: default mode, SAL: salience, CEN: central executive, CAN: central autonomic, ERN: emotion regulation, OCC: occipital.

Brain Regions.

SupFG: superior frontal gyrus, SupCirInS: superior segment of the circular sulcus of the insula, PosCG: postcentral gyrus, IntPS_TrPS: intraparietal sulcus (interparietal sulcus) and transverse parietal sulci, InfTG: inferior temporal gyrus, SupTGLp: lateral aspect of the superior temporal gyrus, SupFS: superior frontal sulcus, PaCL/S: paracentral lobule and sulcus, Thal: thalamus, ATrCoS: anterior transverse collateral sulcus, RG: straight gyrus (gyrus rectus), ACgG_S: anterior part of the cingulate gyrus and sulcus, InfCirInS: inferior segment of the circular sulcus of the insula; CoS_LinS: medial occipito-temporal sulcus (collateral sulcus) and lingual sulcus.

Clinical Variables.

ETI: early traumatic inventory; BMI: body mass index, PSS: Perceived Stress Scale, HADS: Hospital Anxiety and Depression Scale.

In the High ETI group, significant negative associations with key regions of the salience (superior segment of the circular sulcus of the insula), emotion regulation (anterior part of the cingulate gyrus and sulcus), central autonomic (gyrus rectus [straight gyrus]), default mode (inferior temporal gyrus, anterior transverse collateral sulcus), and occipital (medial occipito-temporal sulcus [collateral sulcus]) networks were found with malate and glutamate gamma-methyl ester. Glutamate gamma-methyl ester and 5-oxoproline were negatively associated with symptoms of both anxiety and depression, while urate was negatively associated with symptoms of depression. Connectivity between the sensorimotor and default model networks was positively associated with perceived stress. In addition, sensorimotor networks were negatively correlated with BMI, whereas default mode and central autonomic networks were positively correlated with BMI.

In the Low ETI group, significant negative correlations were found between the connectivity of regions in the sensorimotor (paracentral lobule and sulcus, thalamus, and inferior segment of the circular sulcus of the insula) and emotion regulation (anterior part of the cingulate gyrus and sulcus) networks with glutamate gamma-methyl ester, 5-oxoproline, and urate. However, positive correlations between connectivity in regions of the sensorimotor (postcentral gyrus) and central executive (intraparietal sulcus (interparietal sulcus) and transverse parietal sulci) were positively associated with perceived stress, and symptoms of anxiety and depression. 5-oxoproline and urate were positively associated with ETI total score, while glutamate gamma-methyl ester and 5-oxoproline were negatively associated with BMI.

## 4 Discussion

The current study aimed to test the hypothesis that a history of ELA is associated with altered brain-gut interactions that impact perceived stress, and symptoms of depression and anxiety in adulthood. Our findings support the notion that ELA can lead to persistent disruptions in the brain-gut system, which may contribute to susceptibility to psychological conditions later into adulthood in response to early life adversity. To our knowledge, this is the first study to comprehensively link adversity during childhood to later-life alterations in intestinal metabolites and functional brain connectivity in humans.

### 4.1 Early life adversity is associated with adult gut metabolites

We identified four fecal metabolites – urate, malate, glutamate gamma-methyl ester, and 5-oxoproline – as significantly negatively correlated with a history of ELA. Although a clear understanding of the role of these metabolites in humans is limited, previous preclinical investigations demonstrate that some of them display sensitivity to environmental disruptors and disease (Ilievski et al., 2016), as well as to microbiome alterations (Donohoe et al., 2011) (Donohoe et al., 2011)^,^ (Scheperjans et al., 2015). In particular, high levels of urate in human plasma have been related to protection against Parkinson's disease (Weisskopf et al., 2007) and associated with a microbial enterotype dominated by *Prevotella* (Scheperjans et al., 2015). Conversely, in a sample of pregnant women, early adversity correlated positively with *Prevotella* (Hantsoo et al., 2019). In addition, 5-oxoproline was increased in the livers of mice transplanted with microbiota from patients with major depressive disorder (Li et al., 2018), while levels were decreased in the serum of rats treated with antibiotics (Behr et al., 2017), suggesting a role for the gut microbiota in the regulation of the metabolite.

One potential pathway by which these metabolites may mediate the relationship between ELA, brain connectivity, and mood is via regulation of oxidative stress. ELA has been previously linked to oxidative stress and cellular aging (Schiavone et al., 2015). In a sample of healthy women, oxidative stress index was positively associated with perceived stress and telomere length (Epel et al., 2004). Similarly, a history of childhood maltreatment predicted shorter telomeres (Tyrka et al., 2016) and greater mitochondrial DNA copies (Tyrka et al., 2016), a marker of oxidative damage, in healthy adults. Notably, the four metabolites of interest have previously been implicated in and described within the context of oxidative stress in animal models (Wu et al., 2008; Randhawa et al., 2014; Kumar and A.K., 2012). In particular, 5-oxoproline was reduced in aged rats, and rescued by probiotic treatment, acting as a gut-targeted antioxidant (Hor et al., 2019). In this way, disruptions in the four metabolites may play a role in ELA-related brain network alterations that are mediated by oxidative stress pathways.

An alternative, although potentially related mechanism, is supported by the metabolites being intimately involved in the metabolism of glutamate and related compounds. Glutamate gamma-methyl ester is a metabolite of glutamate (Tsuge et al., 2017), while 5-oxoproline is a precursor and closely-related analogue of glutamate (Kumar and A.K., 2012). Furthermore, 5-oxoproline plays a critical role in glutamate clearance, by stimulating glutamate transport from the brain and inhibiting its uptake by endothelial cells of the blood-brain barrier (Hawkins et al., 2006a). The observed reduction in 5-oxoproline may therefore interfere with CNS clearance of glutamate, which at increased concentrations can be particularly excitotoxic (Dong et al., 2009) in those with a history of high ELA. Additionally, a role for urate-induced, astrocyte-mediated protection against excitotoxicity has been reported *in vitro* (Du et al., 2007). A reduction in these metabolites may lower the threshold for cytotoxicity while simultaneously increasing CNS concentrations of glutamate, thereby increasing the risk for excitotoxity and cell death.

### 4.2 Early life adversity is associated with adult brain functional connectivity

Many types of ELA have previously been associated with altered brain structure and connectivity, including amygdala, prefrontal, limbic, hippocampal, and striatal regions (Hodel et al., 2015; Miskovic et al., 2010; Gee et al., 2013). Here, we identify additional brain regions wherein connectivity was significantly correlated with greater ELA scores, which may explain the relationship between early life adversity and negative psychological outcomes later in life. In particular, we report reduced connectivity of the precuneus, a default mode network region critical for aspects of social cognition (Li et al., 2014), and self-consciousness and interpretation (Cavanna and Trimble, 2006), which may point to altered evaluation of self and others underlying anxious feelings. Indeed, default mode efficiency is negatively correlated with anxiety in young adults (Tao et al., 2015), and default mode connectivity relates to responsiveness during anxiety learning (Tao et al., 2015) as well as being heavily implicated in depressive symptoms (Brakowski et al., 2017). Additionally, our findings of decreased connectivity involving emotion regulation networks such as the anterior cingulate cortex, which is involved in conflict monitoring (Kerns et al., 2004) and emotional and cognitive attention (Bush et al., 2000), and increased connectivity of the insula, a key region in the salience network (Menon and Uddin, 2010), may suggest modified ability to regulate emotional responses. We report increased connectivity of frontal and parietal sensorimotor regions, and central executive and autonomic areas, which is consistent with a meta-analysis implicating executive control, salience, and sensorimotor networks in anxiety (Xu et al., 2019).

The fact that ELA disrupts many regions involved in cognitive and emotional processes, which are highly vulnerable to persistent deleterious effects of ELA (Pechtel and Pizzagalli, 2011), may present a neurological basis underlying our finding that early adversity correlates with later-life stress and anxiety. Similarly, measures of centrality and segregation in brain regions implicated in emotion and salience reportedly correlate with ELA (Gupta et al., 2017b), suggesting that these regions may contribute to psychological manifestations of early trauma. This potential interaction is further supported by findings that functional connectivity of regions, including the amygdala, putamen, and middle frontal gyrus, as well as regions we also identified, such as the middle temporal and superior frontal gyri, differentiated patients with generalized anxiety from healthy controls (Qiao et al., 2017).

### 4.3 A history of early life adversity correlates with alterations in the brain-gut-microbiome system and mood symptoms

We identified significant relationships between fecal metabolites and altered functional brain connectivity measures involving, most notably, the sensorimotor and default mode networks, which have been implicated in both anxiety (Xu et al., 2019) and depression (Brakowski et al., 2017). Previous work has underscored associations between the brain-gut axis and psychological outcomes across the lifetime. Gut-targeted probiotic treatment in healthy adults was sufficient to reduce resting state connectivity in somatosensory and insular areas during an emotional attention task (Tillisch et al., 2013) and to increase prefrontal cortical activity and reduce induced stress (Allen et al., 2016). Connectivity of reward regions has been related to microbiome-derived indole metabolites and anxiety and food addiction outcomes in adults (Osadchiy et al., 2018). In addition, connectivity of regions involved in salience, emotion regulation, and sensorimotor function correlated with microbial diversity and cognitive outcomes in infants (Gao et al., 2019). Interestingly, in pilot analyses, we have also observed relationships between stress and gut health: in particular, we find that increased stress reactivity is associated with a four-fold higher flare frequency in ulcerative colitis patients, and a similar effect in patients with irritable bowel syndrome, both of which are associated with a history of ELA and psychiatric comorbidities. Findings such as these suggest an interaction between psychological well-being and gut microbiome status, both in healthy and in disease populations.

While other studies have found relationships between early adversity, microbial diversity or taxonomic relative abundances, and current stress and anxiety (Callaghan et al., 2020; Hantsoo et al., 2019), we did not see any difference in diversity or relative abundances in our cohort. However, we observe a change in functional output, suggesting that while the levels of microbes are comparable, something about their functional potential is being altered by early adversity. Gut microbial metabolites may influence brain network connectivity through both direct and indirect mechanisms. While 5-oxoproline decreases entry of amino acids into the brain by interacting with transporters (Hawkins et al., 2006b), urate is capable of passing across the blood-brain barrier and acts as a pro-inflammatory agent (Shao et al., 2016). However, since the metabolites in this study were measured in feces rather than serum, whether these metabolites may have any direct access to the brain remains unclear. Alternatively, these metabolites may act indirectly via vagal afferent nerve pathways (Bartolomei et al., 2016; Cao et al., 2017), which in turn may contribute to the observed changes in functional connectivity.

Not only ELA, but other negative emotional and physiological states have the potential to interact with the brain-gut axis as well. We show that symptoms of anxiety and depression and BMI correlate significantly with urate, glutamate gamma-methyl ester, and 5-oxoproline, that these scales as well as current stress relate significantly to brain functional connectivity of sensorimotor, central executive, default mode, and central autonomic regions, and that subsets of these networks, in addition to salience, emotion regulation, and occipital, correlate significantly with the metabolites. These relationships are of significance due to the potential functional influence of altered sensory modalities and orbitofrontal cortex function, which are critical for decision-making (Bechara et al., 2000), on negative psychological states. Similar findings have been reported in the context of food addiction, with amygdala circuitry and the gut microbiota-derived indole skatole correlating with higher food addiction scores (Osadchiy et al., 2018). Additionally, stress-related disorders such as PTSD have been related to altered connectivity in the hippocampus (Chen and Etkin, 2013) as well as in amygdala-insula circuits (Rabinak et al., 2011), and acute stress has been related to metabolites, with increased CSF homovanillic acid correlating with induced symptoms in PTSD patients (Geracioti et al., 2013). However, these past studies do not decouple contributions of past adversity from current experiences of stress and anxiety.

### 4.4 Future directions

Limitations of this study include its use of retrospective self-reporting through standardized surveys, which can introduce potential bias into the results and may potentially lead to reduced reliability of the findings. This limitation includes the self-reporting for assessment of menstrual phase and menopause status, which future studies will need to address more accurately by measuring female sex hormone levels especially when investigating sex differences. Critically, our measure of early adversity is not temporally specific, but rather covers the broad period of time from birth to 18 years of age. Future research is warranted to refine this time window to examine relationships between brain-gut phenotypes and ELA during more specific critical periods during development. Secondly, our reported microbial and metabolite findings are derived from fecal samples, which include both microbiota- and host-derived metabolites, and do not necessarily give insight into tissue levels within the CNS. Furthermore, we analyzed only a single fecal sample per participant, although the microbiome and metabolome have been reported to be relatively stable across adulthood (Lozupone et al., 2012; Yousri et al., 2014). We chose to focus on gut metabolites as opposed to microbial community composition as most brain-gut interactions are mediated by microbiota products (such as metabolites) rather than an intrinsic characteristic of the particular microbe itself (such as lipopolysaccharide) (Osadchiy et al., 2019c). Previous high-quality studies (Wu et al., 2011; Tanes et al., 2021) have underscored the important role of dietary fiber in modulating the gut microbiota and gut-microbiota derived fecal metabolites. Although we controlled for the type of diet consumed in our analysis, we did not quantify the fiber content of our participants’ diets, which may confound our results.

Although the associations between ELA and the brain-gut axis were evident, our sample consisted of participants with a relatively “healthy” status, where anyone with clinical level symptoms of anxiety or depression were excluded from the study. We selected these participants with the intent to highlight and isolate the negative outcomes associated with ELA that are distinct from any effects from overt neurological disease. Future studies that expand upon reported brain-gut phenotypes in individuals with ELA-associated conditions, such as anxiety and depression, are of interest. Finally, while the results from this study reveal novel associations between ELA and later-life alterations in microbiome-related metabolites and functional brain connectivity, the cross-sectional study design precludes the ability to make causal inferences. Longitudinal studies in humans would help to strengthen correlations and shed light on the timing of interactions between early trauma and altered metabolites and functional brain connectivity.

Although we control for BMI in our final analysis exploring the relationship between ELA, metabolites, and clinical measures, it is important to highlight the positive association between ELA and BMI we identify here – a relationship that has also been confirmed in previous work (Fuemmeler et al., 2009; Osadchiy et al., 2019d). Obesity is increasingly understood as a brain-gut-microbiome disorder, with mechanisms similar to those we identify in this present work with respect to anxiety and depression. Future investigations may benefit from exploring the intersection between ELA and BMI on these clinical measures within the context of brain-gut interactions (Gupta et al., 2020).

### 4.5 Clinical implications and conclusions

Our findings in human subjects with a history of ELA demonstrate associations that may support the hypothesis that traumatic experiences during critical periods of brain and gut development shape long-term changes in brain-gut interactions. We suggest that this may occur via the well characterized effect of ELA on brain networks involved in emotion regulation and autonomic nervous system output to potentially alter gut microbial function, in the form of microbially-modulated metabolites. The observed dysregulation of glutamate pathways may result in excitotoxicity and oxidative stress, disrupting neural circuit assembly and existing brain network connectivity, and increasing the risk of developing anxiety and depression. Overall, findings from the study provide clinical evidence of brain-gut alterations in response to ELA, and further form a solid foundation upon which to assess potential roles for the microbiome in mediating adverse effects of ELA on brain development and later-life behavior.

## CRediT authorship contribution statement

**Elena J.L. Coley:** analysis, drafting of the manuscript, critical revision of the manuscript for important intellectual content. **Emeran A. Mayer:** funding, study concept and design, critical revision of the manuscript for important intellectual content, study supervision. **Vadim Osadchiy:** data interpretation, critical revision of the manuscript for important intellectual content. **Zixi Chen:** statistical analysis. **Vishvak Subramanyam:** statistical analysis. **Yurui Zhang:** statistical analysis. **Elaine Y. Hsiao:** data interpretation, critical revision of the manuscript for important intellectual content. **Kan Gao:** statistical analysis. **Ravi Bhatt:** statistical analysis. **Tien Dong:** data interpretation, critical revision of the manuscript for important intellectual content. **Priten Vora:** statistical analysis. **Bruce Naliboff:** data interpretation, critical revision of the manuscript for important intellectual content. **Jonathan P. Jacobs:** data interpretation, critical revision of the manuscript for important intellectual content. **Arpana Gupta:** funding, study concept and design, analysis and interpretation of data, drafting of the manuscript, critical revision of the manuscript for important intellectual content, statistical analysis, technical support, study supervision.

## Declaration of competing interest

AG is scientific advisor to Yamaha. EAM is a scientific advisory board member of Danone, Axial Biotherapeutics, Viome, Amare, Mahana Therapeutics, Pendulum, Bloom Biosciences, APC Microbiome Ireland. EYH is former co-founder of Axial Biotherapeutics, co-founder of Bloom Biosciences and Purpose Bio, and scientific advisor to Holobiome.

All other authors have nothing to disclose.

The results of this manuscript are part of a U.S. provisional patent application no. 63/107,998.

The authors would like to acknowledge the following for feedback and assistance with the preparation of the manuscript: Cathy Liu, Ziheng Qu.

## Data Availability

Data will be made available on request.
