# Supplementary figures and images for: Early life adversity predicts brain-gut alterations associated with increased stress and mood
Source: Neurobiol Stress. 2021 May 25;15:100348. doi: 10.1016/j.ynstr.2021.100348 (PMC8170500; doi:10.1016/j.ynstr.2021.100348)

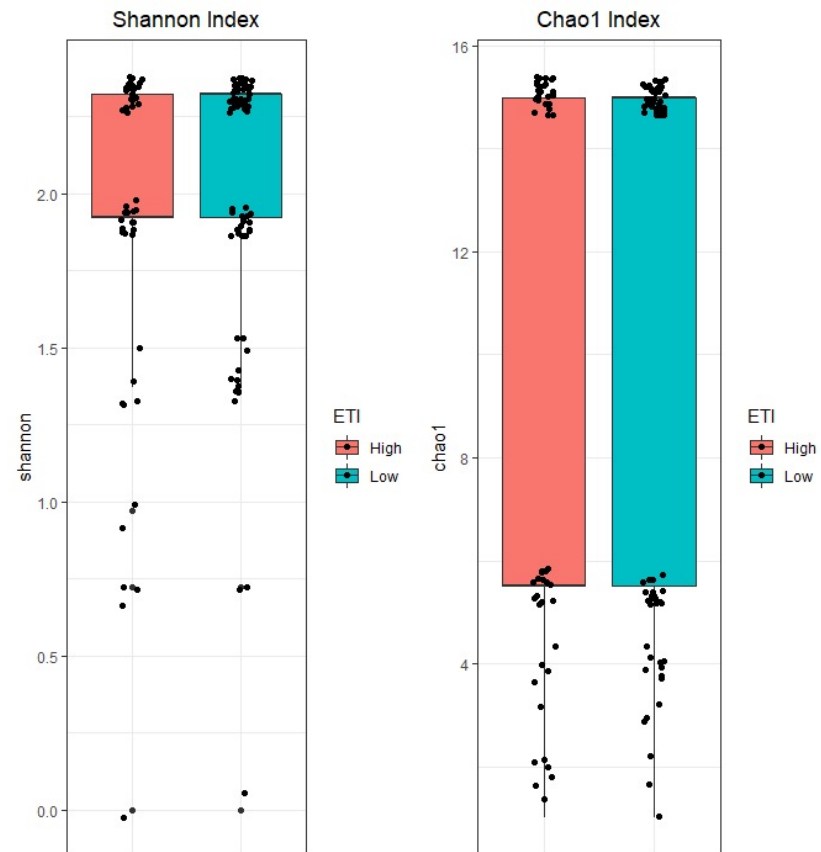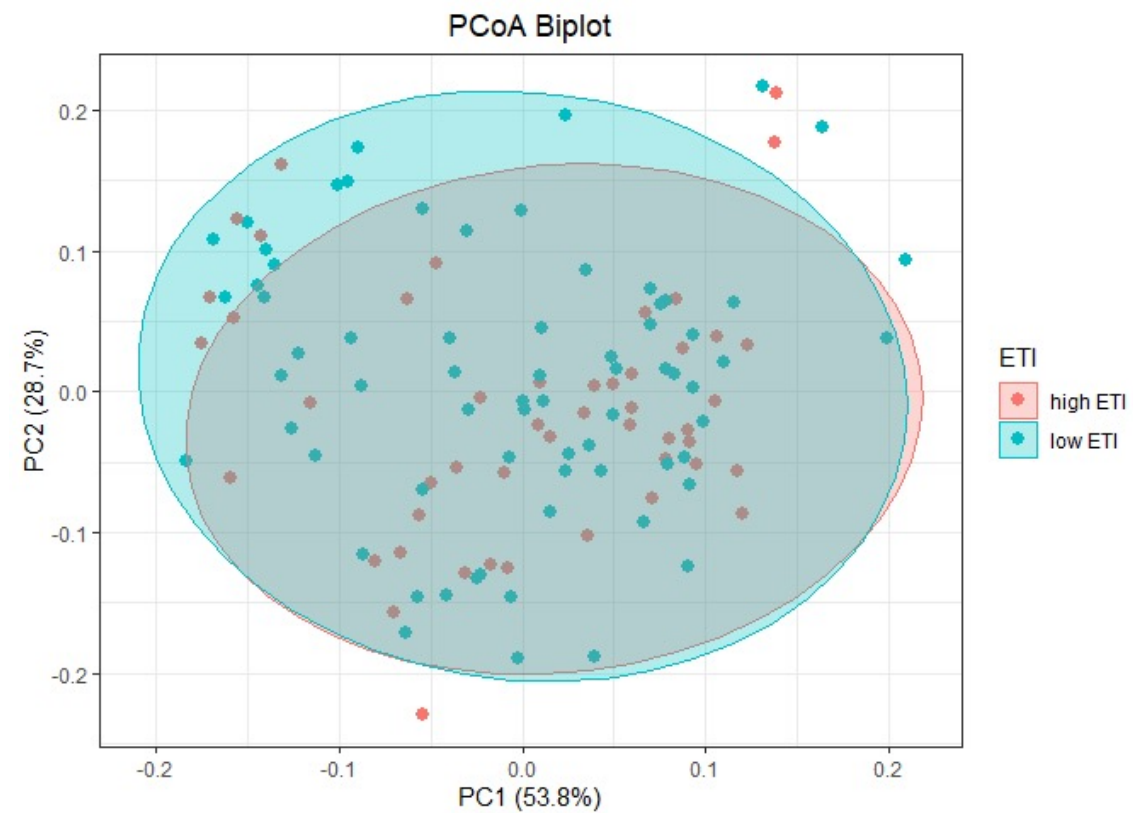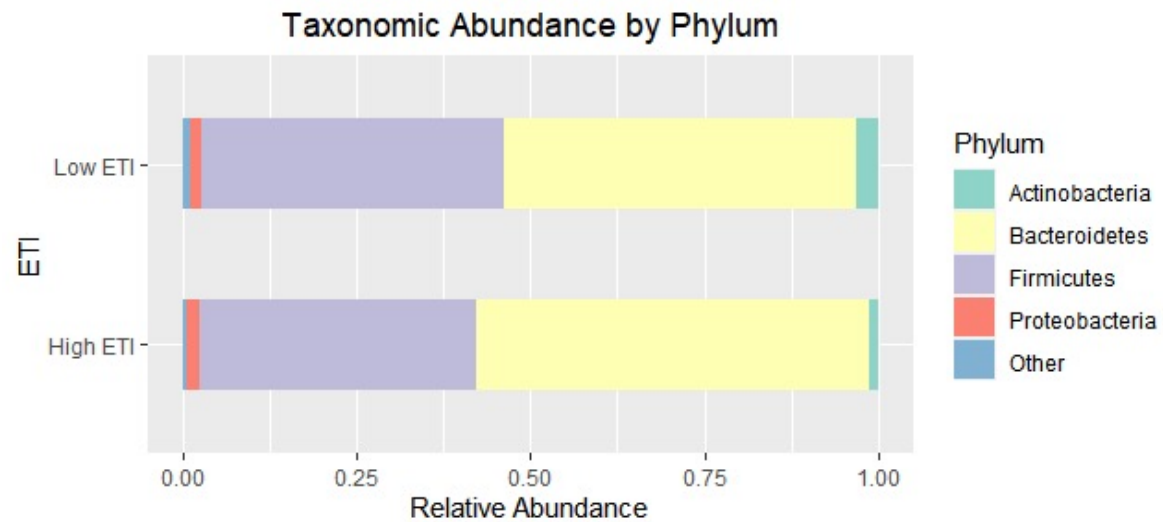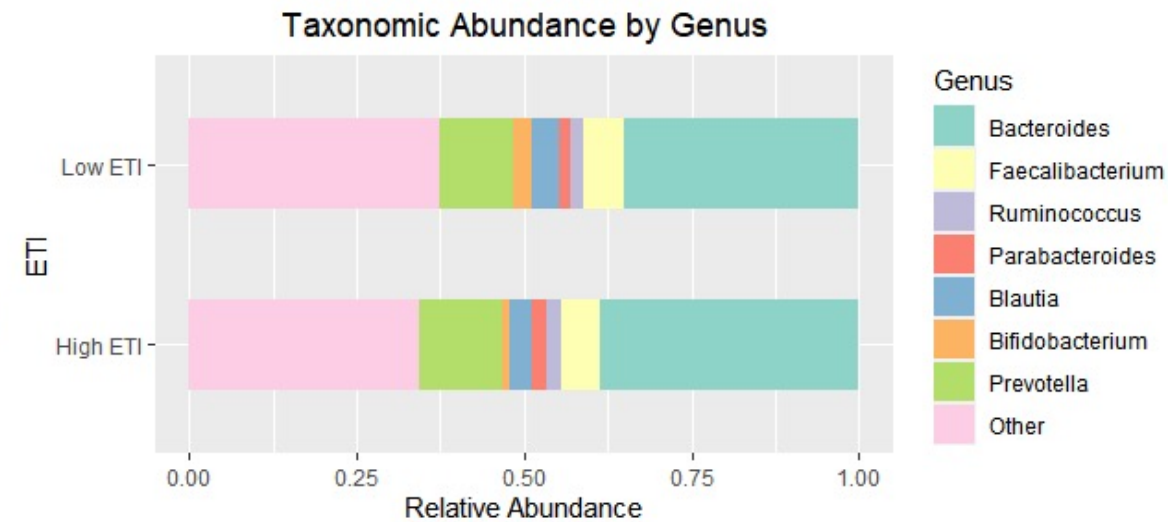

Supplement: Multimedia component 1 [file mmc1.pdf]
